# Cell cycle pathway alterations predict outcomes post-liver transplantation for hepatocellular carcinoma

**DOI:** 10.3389/frtra.2026.1758576

**Published:** 2026-04-10

**Authors:** Ashton A. Connor, Mahinur Mattohti, Sudha Kodali, Maen Abdelrahim, Ahmed Elaileh, Samar Semaan, Youssef Dib, Khush Patel, Jason Todd, Urmi Sengupta, Constance M. Mobley, Caroline J. Simon, Yee Lee Cheah, Ashish Saharia, Mary R. Schwartz, Sadhna Dhingra, Jessica S. Thomas, Randall J. Olsen, Linda W. Moore, Kirk Heyne, Xian C. Li, David W. Victor, Timothy E. Newhook, Ahmed O. Kaseb, Jean-Nicolas Vauthey, A. Osama Gaber, R. Mark Ghobrial

**Affiliations:** 1 Department of Surgery, Houston Methodist Hospital, Houston, TX, United States; 2 JC Walter Jr Transplant Center, Houston Methodist Hospital, Houston, TX, United States; 3 Department of Surgery, Weill Cornell Medical College, New York, NY, United States; 4 Immunobiology and Transplant Science Center, Houston Methodist Research Institute, Houston Methodist Hospital, Houston, TX, United States; 5 Sherrie and Alan Conover Center for Liver Disease and Transplantation, Houston Methodist Hospital, Houston, TX, United States; 6 Department of Medicine, Weill Cornell Medical College, New York, NY, United States; 7 Department of Medical Oncology, Houston Methodist Cancer Center, Houston, TX, United States; 8 Cockrell Center Phase 1 Unit, Cockrell Center for Advanced Therapeutics, Houston Methodist Hospital, Houston, TX, United States; 9 Department of Pathology and Genomic Medicine, Houston Methodist Hospital, Houston, TX, United States; 10 The University of Texas MD Anderson Cancer Center, Houston, TX, United States

**Keywords:** biomarkers, cell cycle pathway, hepatocellular carcinoma, liver transplantation, molecular profiling

## Abstract

**Background:**

Hepatocellular carcinoma (HCC) incidence and mortality are rising. Liver transplantation (LT) offers the best outcomes, but current tumor size- and number-based selection criteria restrict access. Molecular profiling may better reflect tumor biology and guide precision-based selection strategies.

**Methods:**

This prospective single-center study included patients with HCC who had undergone LT between 11 November 2016 and 4 April 2023 with sufficient tumor cellularity in explanted livers. Tumor DNA was subjected to targeted sequencing for 38 genes, with the results returned to clinicians. Altered genes were grouped into HCC-relevant signaling pathways. Outcomes included post-LT overall survival (OS), recurrence-free survival (RFS), and recurrence sites. The Cancer Genome Atlas (TCGA) HCC cohort was used for validation.

**Results:**

Among 1,103 LT recipients, 261 were for HCC and 91 underwent sequencing. Most patients were male (*n* = 68), white (*n* = 56), and hepatitis C positive (*n* = 34). The median tumor size was 3 cm (IQR 1.8–4.5), the number was 1 (IQR 1–3), and the follow-up time was 1,982 days. Of the 36 unique mutations found across 11 genes, six were potentially actionable. Cell cycle pathway alterations (*n* = 14) were prognostic for worse OS (3-year 90.9% without vs. 62.5% with alterations) and RFS in uni- and multivariable models. Recurrences were more common in the liver and lungs with cell cycle alterations (*p* < 0.05).

**Conclusion:**

Post-LT molecular profiling of HCC reveals tumor-specific alterations associated with outcomes, supporting the incorporation of tumor biology into future LT selection criteria.

## 1 Introduction

Hepatocellular carcinoma (HCC) is the third leading cause of cancer-related mortality globally, responsible for approximately 700,000 deaths every year (1, 2). Liver transplantation (LT) offers the best long-term outcomes (3, 4). Eligibility based on tumor size and number enables the selection of patients at low risk of recurrence and death after transplant (5). However, patients exceeding burden-based criteria can have excellent post-LT survival (6). Markers of tumor biology, such as neoadjuvant therapy response, have been suggested to better inform selection (6–9).

HCC biology is described in several series, including the Cancer Genome Atlas (TCGA), the International Cancer Genome Consortium, and the Chinese Liver Cancer Atlas (1–4, 10–20). These studies identified altered driver genes, signaling pathways, and potential therapeutic targets. Translationally, one series identified HCC molecular features predicting response to immunotherapy, and several series identified biomarkers of tyrosine kinase inhibitor susceptibility (21–23). However, most studies were retrospective and did not involve LT. Thus, integration of HCC biology into peritransplant care pathways remains an elusive proposition. Concurrently, studies in secondary hepatic malignancies have shown that somatic alterations grouped by signaling pathways can be more informative of tumor biology than driver gene mutations alone (24).

The purpose of this study is to describe molecular profiling of HCC in patients who had undergone LT. Molecular alterations at the pathway level were associated with clinical and pathologic features, including post-LT overall survival (OS), recurrence-free survival (RFS), and sites of recurrence. This suggests how HCC biology may be integrated into LT care.

## 2 Materials and methods

### 2.1 Clinical variables

LT patients with HCC who had undergone tumor molecular profiling at the authors' institution between 1 November 2016, and 30 April 2023 were retrospectively identified from a prospectively maintained database. Clinical and pathological variables were extracted from electronic medical records. Donor graft type was always deceased donor whole liver allografts. Post-transplant immunosuppression clinical practice guidelines at our institution call for the use of corticosteroid bolus and taper, tacrolimus with FK goal 5−7, and mycophenolate. Research was conducted under the center's Institutional Review Board Protocol Pro00000587.

### 2.2 Molecular profiling

Inclusion criteria for molecular profiling required histologically diagnosed HCC on explanted liver pathology with sufficient tumor tissue to undergo next-generation sequencing (NGS). The decision to perform sequencing was left to the discretion of the patient's treatment team.

Formalin-fixed, paraffin-embedded explanted liver specimens from LT recipients were subjected to standard macroscopic and histopathologic assessment. HCCs of sufficient cellularity for molecular profiling were identified prospectively. Tumor genomic DNA was extracted from unstained slides and subjected to targeted NGS testing using either a 44- or a 50-gene solid tumor mutation panel.

The 50-gene NGS panel was performed in the Molecular Diagnostics Laboratory at Houston Methodist Hospital (Houston, TX, USA). The 44-gene NGS panel was performed by ARUP Laboratories (Salt Lake City, UT, USA) and used only from March 2020 to November 2021. Overlap between the two panels consisted of 38 genes. Both panels utilized the Ion Proton System with Torrent Server (ThermoFisher Scientific) to identify somatic mutations. The resulting short read sequences were aligned to the human genome (hg19) and annotated using the Houston Methodist Variant Viewer software (25, 26). Variant calling criteria were as follows: a minimum read depth of 100, non-synonymous base pair substitutions or insertions/deletions, an allelic frequency of >10%, somatic origin, and variants previously reported in human cancers. Alignment and annotation adhered to the National Cancer Institute Genomic Data Commons guidelines (27). The variants were further annotated for potential actionability using OncoKB (28). Gene-level mutations were organized into signaling pathways previously identified in HCC (1, 29).

### 2.3 Validation cohort

Clinical and mutation data for the TCGA Hepatocellular Carcinoma project were downloaded from cBioPortal (1, 30–32). Mutations were filtered by impact (“high” or “moderate”), clinical significance (“pathogenic,” “likely pathogenic”), and the 38 genes sequenced in the discovery cohort. The mutations were assigned to pathways.

### 2.5 Statistical analysis

Associations of clinical and pathologic features of LT recipients and donors with molecular features were determined by performing Fisher's exact or chi-squared tests for categorical variables and Kolmogrov–Smirnov or *t*-tests for continuous variables. Uni- and multivariable Cox proportional hazards models determined the association of variables with OS and RFS. Stepwise backward elimination regression was used for selection of variables in final multivariable Cox models. ANOVA was used to compare the fit of nested multivariable Cox proportional hazard models to outcomes data. Analyses were performed using R version 4.4.1 (R Foundation for Statistical Computing, Vienna, Austria). A cutoff *p*-value of 0.05 determined statistical significance.

## 3 Results

### 3.1 Patient selection and features

During the study period, 1,103 LTs were performed, of which 261 were for HCC. A total of 91 patients had explanted HCC tumors that were subjected to targeted sequencing. Patients with sequenced (*n* = 91) vs. non-sequenced tumors (*n* = 170) were well matched with regard to most peritransplant parameters, including age and functional status at transplant, underlying causes of liver disease, educational level, and balance between public and private payers (Supplementary Table S1). Tumors less often underwent sequencing post-LT during the period of the COVID-19 pandemic (2020−2021). By explant pathology, HCC tumors that underwent sequencing were significantly although slightly larger in maximum diameter [median 3.0 cm (IQR 1.8−4.5 cm) vs. 2.4 cm (IQR 1.2−3.5 cm), *p* = 0.002], were greater in number [median 1 lesion (IQR 1−3 lesions) vs. 1 lesion (IQR 1−2 lesions), *p* = 0.024], had less tumor necrosis [50% (IQR 5−78%) vs. 98% (IQR 67−100%), *p* < 0.001], had more frequent lymphovascular invasion (LVI) [18.7% vs. 7.7%, *p* = 0.015], and had a greater TNM stage [*p* < 0.001], as expected from the inclusion criteria of sufficient tumor cellularity for sequencing. Most importantly, post-transplant outcomes of OS and RFS were not significantly different between sequenced and non-sequenced tumors (*p* = 0.974 and 0.205, respectively).

The 91 patients who had undergone HCC sequencing (Table 1) were predominantly male (*n* = 67, 73.6%), white (*n* = 55, 60.4%), and Hispanic (*n* = 27, 29.7%). Most patients had cirrhosis (*n* = 86, 94.5%). The most common etiologies of liver disease were chronic viral hepatitis (*n* = 35, 38.5%) and metabolic-associated steatohepatitis (*n* = 16, 17.6%). The median waitlist time was 308 (IQR 118−470) days. Most patients received some pretransplant liver-directed therapy (*n* = 70, 76.9%), most commonly, transarterial chemoembolization (TACE; *n* = 42, 46.2%). A minority of patients received pretransplant systemic therapy (*n* = 14, 15.4%), most commonly a tyrosine kinase inhibitor (*n* = 10, 11%). On explant pathology, HCC tumors were a mixture of early and locally advanced stages, with 45 (49.5%) multifocal cancers with a median maximum size of 3 (IQR, 1.8−4.5) cm and 17 (18.7%) with LVI. Post-transplant recurrences were identified in 13 (14.3%) patients, with the initial site of recurrence most commonly in the liver (*n* = 10, 11%). By the last follow-up, it was found that 23 (25.3%) patients had died, most frequently due to cancer (*n* = 7) or infection (*n* = 5).

**Table 1:** Demographics, clinical characteristics, and outcomes of patients who had undergone liver transplantation for hepatocellular carcinoma stratified by cell cycle pathway alterations.

Variable		Total (*N* = 91)	Cell cycle pathway intact (*N* = 77)	Cell cycle pathway altered (*N* = 14)	*p*-Value
Donor features					
Donor type, *N* (%)	DBD	84 (92.3)	71 (92.2)	13 (92.9)	1.000
	DCD	7 (7.7)	6 (7.8)	1 (7.1)	
Recipient features at liver transplant					
Age, med (IQR)		62.0 (57.0–67.0)	62.0 (56.0–66.0)	63.5 (59.0–68.0)	0.134
Biological sex, *N* (%)	Male	67 (73.6)	58 (75.3)	9 (64.3)	0.594
BMI, med (IQR)		28.5 (25.8–33.5)	28.5 (25.8–33.7)	28.9 (26.4–33.1)	0.721
MELD at transplant, med (IQR)		17.0 (9.0–31.0)	17.0 (9.0–31.0)	18.5 (10.0–34.0)	0.512
Race/ethnicity, *N* (%)	Non-Hispanic White	55 (60.4)	48 (62.3)	7 (50)	0.610
	Non-Hispanic Black	3 (3.3)	3 (3.9)	0 (0)	
	Non-Hispanic Asian	6 (6.6)	5 (6.5)	1 (7.1)	
	Hispanic	27 (29.7)	21 (27.3)	6 (42.9)	
Primary liver disease diagnosis, *N* (%)	Autoimmune	6 (6.6)	3 (3.9)	3 (21.4)	0.048
	Metabolic	16 (17.6)	15 (19.5)	1 (7.1)	
	Viral	35 (38.5)	31 (40.3)	4 (28.6)	
	Toxic	15 (16.5)	14 (18.2)	1 (7.1)	
	Other	19 (20.9)	14 (18.2)	5 (35.7)	
Pre-transplant adjunctive cancer therapy					
Hepatectomy, *N* (%)	Yes	2 (2.2)	1 (1.3)	1 (7.1)	0.703
Liver-directed therapy—any, *N* (%)	Yes	70 (76.9)	60 (77.9)	10 (71.4)	0.853
Systemic therapy—any, *N* (%)	Yes	14 (15.4)	11 (14.3)	3 (21.4)	0.780
Post-transplant outcomes					
Post-transplant recurrence, *N* (%)	Yes	13 (14.3)	8 (10.4)	5 (35.7)	0.038
Recurrence-free survival, med (IQR)		1,414.0 (578.0–1,897.0)	1,459.0 (632.0–1,869.0)	1,020.0 (407.0–2,000.0)	0.362
Initial recurrence site, *N* (%)	Liver	10 (11.0)	5 (6.5)	5 (35.7)	0.006
	Lung	5 (5.5)	2 (2.6)	3 (21.4)	0.027
Post-transplant patient survival, med (IQR)		1,982.0 (1,139.5–2,516.5)	2,012.0 (1,233.0–2,457.0)	1,040.0 (932.0–2,576.0)	
Patient cause of death, *N* (%)	Cancer	7 (38.9)	4 (30.8)	3 (60)	0.627
	Cardiac	1 (5.6)	1 (7.7)	0 (0)	
	Infection	5 (27.8)	3 (23.1)	2 (40)	
	Multiple Organ Failure	1 (5.6)	1 (7.7)	0 (0)	
	Renal	1 (5.6)	1 (7.7)	0 (0)	
	Respiratory	3 (16.7)	3 (23.1)	0 (0)	
Explanted tumor features					
Focality, *N* (%)	Multifocal	45 (49.5)	37 (48.1)	8 (57.1)	0.737
Number, med (IQR)		1.0 (1.0–3.0)	1.0 (1.0–3.0)	2.0 (1.0–3.0)	0.920
Maximum diameter in cm, med (IQR)		3.0 (1.8–4.5)	2.9 (1.8–4.1)	4.0 (3.3–4.8)	0.050
Histological grade, *N* (%)	G1	24 (26.7)	20 (26.3)	4 (28.6)	0.033
	G2	57 (63.3)	51 (67.1)	6 (42.9)	
	G3	9 (10.0)	5 (6.6)	4 (28.6)	
Lymphovascular invasion, *N* (%)	Yes	17 (18.7)	14 (18.2)	3 (21.4)	1.000
Tumor necrosis (%), med (IQR)		50.0 (5.0–78.0)	54.0 (5.0–80.0)	25.0 (5.0–50.0)	0.171
TNM stage, *N* (%)	1	44 (48.4)	38 (49.4)	6 (42.9)	0.594
	2	40 (44.0)	34 (44.2)	6 (42.9)	
	3	7 (7.7)	5 (6.5)	2 (14.3)	
Molecular features					
Sequencing platform, *N* (%)	External	10 (11.0)	8 (10.4)	2 (14.3)	1.000
	Internal	81 (89.0)	69 (89.6)	12 (85.7)	
Tumor cellularity, med (IQR)		70.0 (60.0–80.0)	70.0 (60.0–80.0)	65.0 (55.0–70.0)	0.249
Transplant to pathology time (days), med (IQR)		6.0 (4.0–18.5)	7.0 (4.0–23.0)	5.5 (3.0–7.0)	0.200
Pathology to molecular time (days), med (IQR)		17.0 (14.0–23.0)	16.0 (13.0–22.0)	21.5 (17.0–24.0)	0.013
Transplant to molecular time (days), med (IQR)		27.0 (21.0–43.0)	27.0 (21.0–43.0)	27.5 (23.0–43.0)	0.692
Total reported somatic mutations (per tumor), *N* (%)	0	52 (57.1)	52 (67.5)	0 (0)	<0.001
	1	25 (27.5)	18 (23.4)	7 (50)	
	2	11 (12.1)	5 (6.5)	6 (42.9)	
	3	3 (3.3)	2 (2.6)	1 (7.1)	
Total observed pathway alterations (per tumor), *N* (%)	0	57 (62.6)	57 (74)	0 (0)	<0.001
	1	27 (29.7)	19 (24.7)	8 (57.1)	
	2	7 (7.7)	1 (1.3)	6 (42.9)	

BMI, body mass index; ICU, intensive care unit; HCC, hepatocellular carcinoma; LT, liver transplantation; LRT, locoregional therapy; MELD, model for end-stage liver disease; TACE, transarterial chemoembolization; TARE, transarterial radioembolization.

### 3.2 Molecular profiling

The median time from date of transplantation to molecular reporting was 27 (IQR, 21−43) days. Approximately a third of this time ranged from transplant to pathology reporting and two-thirds from pathology to molecular reporting (Supplementary Figure S1). No tumor identified in our review failed to undergo sequencing.

### 3.3 Gene-level alterations

**Figure 1:**
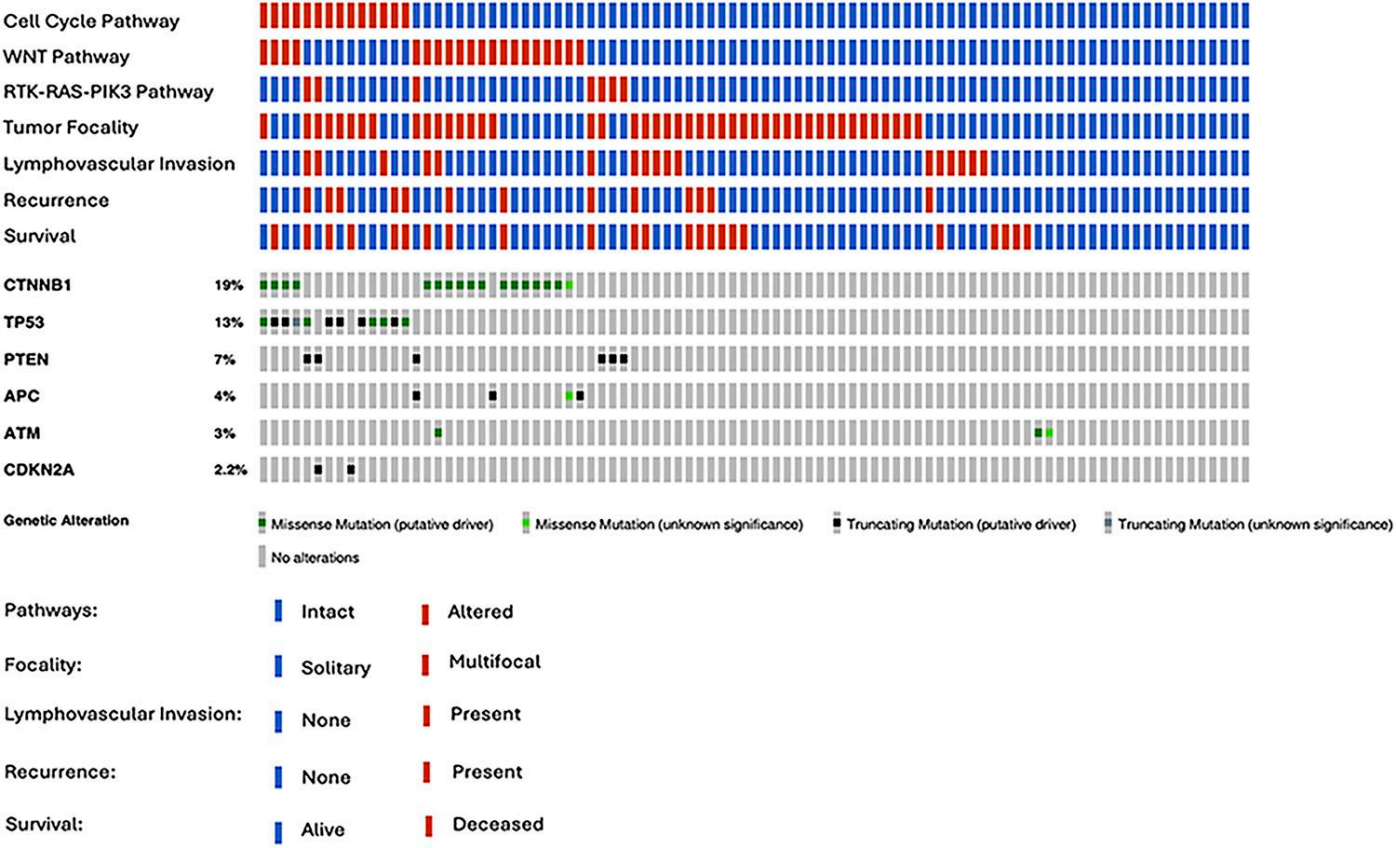
An OncoPrint of (top down) altered gene signaling pathways, pathological and clinical features, and driver gene alterations in 91 patients with hepatocellular carcinoma who had undergone liver transplantation.

A total of 36 unique mutations were identified in 11 genes (Figure 1; Supplementary Table S2), and the mutation types were 22 missenses and 14 truncations. The most frequently altered genes were catenin beta−1 (*CTNNB1*; *n* = 17 patients, 18.7%), tumor protein p53 (*TP53*; *n* = 12, 13.2%), phosphatase and tensin homolog (*PTEN*; *n* = 6, 6.6%), adenomatous polyposis coli (*APC*; *n* = 4, 4.4%), and ataxia–telangiectasia mutated (*ATM*; *n* = 3, 3.3%). The number of carriers per mutation ranged from 1 to 5 and the number of non-synonymous mutations per tumor ranged from 0 to 3. Thirty-nine (42.9%) patients carried at least one gene-level mutation. Tumor cellularity of the sequenced block was reported in 55 (60.4%) cases and was not associated with mutation reporting (all *p* > 0.05).

Nearly all mutations were annotated as “likely oncogenic” in the OncoKB database (Supplementary Table S2); thus, every mutation was assumed equally likely to alter gene function (i.e., of equally weighted pathogenicity) (28). According to OncoKB, the mutations were potentially actionable in four genes altered in 22 patients (Supplementary Table S2), a similar proportion to what has been previously reported in HCC (17, 19, 21). One altered gene, *TP53*, was reported as prognostic (OncoKB level of evidence 1−3), with eight mutations in 12 patients. Three altered genes were reported as predictive of therapeutic response for targeted agents (FDA level of evidence 2−3), with six mutations also in 10 patients. No mutations resulted in changes in patient care. Gene-level alteration in *TP53* was associated with RFS (log-rank 0.0019), similarly to what was reported elsewhere (13), but not with OS (*p* = 0.083).

### 3.4 Pathway alterations

The mutated genes were grouped into signaling pathways previously identified as altered in HCC (1). Alterations were identified in three pathways, namely the RTK-RAS-PIK3 pathway in 7 (7.7%) tumors, the cell cycle pathway in 14 (15.4%) tumors, and the wingless/integrated (WNT) pathway in 20 (22%) tumors (Figure 1). These were not exclusive, with 7 (7.7%) tumors having two altered pathways. The cell cycle pathway includes the *TP53* and *CDKN2A* genes. There were two patients with HCCs with cell cycle pathway alterations that were wild type for *TP53* and had gene-level alterations in *CDKN2A*.

### 3.5 Association of pathways with clinical and pathological characteristics

Peritransplant clinical and pathological features associated with patients and tumors in each altered pathway were investigated. There were no associations with donor features (Table 1, Supplementary Figures S2,S3, Supplementary Table S3). Patients whose HCCs had alterations in the cell cycle pathway more often arose with certain liver disease etiologies, including autoimmune etiologies, and less often with metabolic, toxic, and viral etiologies. On explant pathology, HCCs with cell cycle pathway alterations were of a significantly higher grade and larger size. There was no significant association of pathway alterations with pre-LT locoregional or systemic therapies. Patients with these HCCs were more likely to have the liver and lung as sites of first recurrence. Patients whose HCCs had alterations in the WNT pathway had lower biological MELD scores at transplant, while those with alterations in the RTK-RAS-PIK3 pathway also had a significantly higher grade (Supplementary Table S3).

### 3.6 Association of pathways with post-transplant outcomes

**Figure 2:**
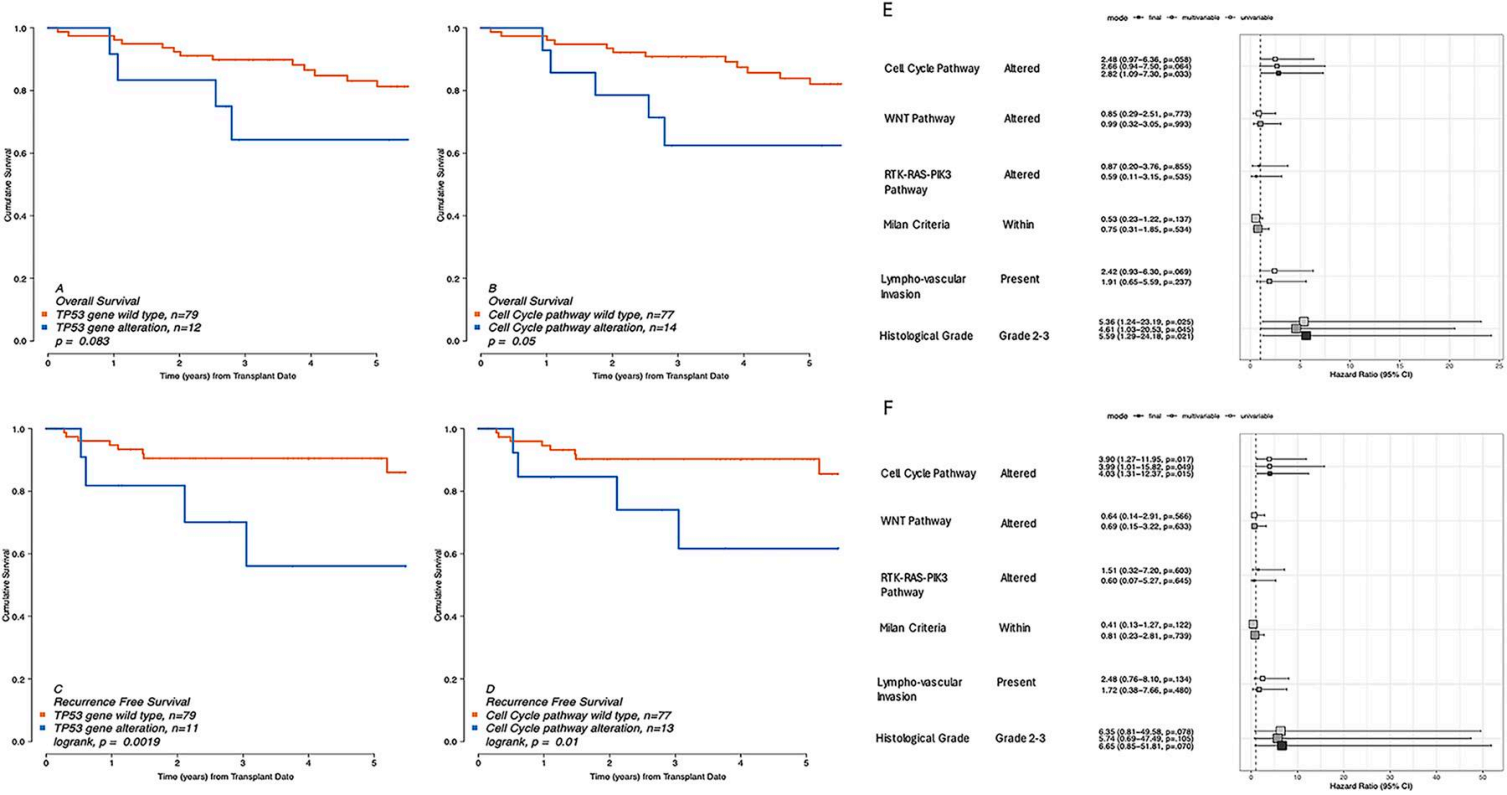
Post-liver transplant outcomes for 91 patients with hepatocellular carcinoma. Overall **(A,B)** and recurrence-free **(C,D)** survival stratified by *TP53*-gene-level mutations **(A,C)** and cell cycle pathway alterations **(B,D)**. Associations of variables in uni- and multivariable Cox proportional hazards models with **(E)** overall and **(F)** recurrence-free survival.

A total of 13 (14.3%) recurrences and 23 (25.3%) deaths were observed in this cohort (Table 1). The median follow-up time was 1982 (1,139.5−2,516.5) days. For the entire cohort, the 1-, 3-, and 5-year post-LT RFS and OS rates were 93.1%, 87.9%, and 86.2% and 96.7%, 86.7%, and 80.7%, respectively. In the 13 patients with recurrence, the median time from transplantation to recurrence was 400 days (IQR 193−771). Cox proportional hazards models were constructed with variables chosen *a priori* based on known risk factors for HCC outcomes (Table 2). Tumor number and size were stratified by the so-called Milan criteria (5). Alterations in the cell cycle pathway were associated with worse RFS and OS in both uni- and multivariable models (Figures 2A–F), more strongly, in fact, than any other included clinical or pathological variables. At the univariable level, the cell cycle pathway, which includes the *TP53* gene, showed a greater association with OS than *TP53* alteration alone (Figures 2A,B). At the multivariable level, models including cell cycle pathway alterations vs. *TP53* gene-level alterations alone provided a significantly better fit to the OS and RFS data (*p* < 0.001 for each by ANOVA). In addition, histology grade was associated with worse outcomes. Neither OS nor RFS was associated with total gene-level or pathway-level mutations (Supplementary Figure S4), as also reported in previously published series (23).

**Table 2:** Uni- and multivariable Cox proportional hazards models for recurrence-free and overall survival after liver transplantation for hepatocellular carcinoma in 92 patients.

Variable	Strata	Recurrence free survival			Overall survival		
		HR (univariable)	HR (multivariable)	HR (final)	HR (univariable)	HR (multivariable)	HR (final)
Cell cycle pathway	Intact						
	Altered	3.90 (1.27–11.95, *p* = 0.017)	3.99 (1.01–15.82, *p* = 0.049)	4.03 (1.31–12.37, *p* = 0.015)	2.48 (0.97–6.36, *p* = 0.058)	2.66 (0.94–7.50, *p* = 0.064)	2.82 (1.09–7.30, *p* = 0.033)
WNT pathway	Intact						
	Altered	0.64 (0.14–2.91, *p* = 0.566)	0.69 (0.15–3.22, *p* = 0.633)		0.85 (0.29–2.51, *p* = 0.773)	0.99 (0.32–3.05, *p* = 0.993)	
RTK-RAS-PIK3 pathway	Intact						
	Altered	1.51 (0.32–7.20, *p* = 0.603)	0.60 (0.07–5.27, *p* = 0.645)		0.87 (0.20–3.76, *p* = 0.855)	0.59 (0.11–3.15, *p* = 0.535)	
Milan criteria	Beyond						
	Within	0.41 (0.13–1.27, *p* = 0.122)	0.81 (0.23–2.81, *p* = 0.739)		0.53 (0.23–1.22, *p* = 0.137)	0.75 (0.31–1.85, *p* = 0.534)	
Lymphovascular invasion	None						
	Yes	2.48 (0.76–8.10, *p* = 0.134)	1.72 (0.38–7.66, *p* = 0.480)		2.42 (0.93–6.30, *p* = 0.069)	1.91 (0.65–5.59, *p* = 0.237)	
Histology grade	G1						
	G2, and G3	6.35 (0.81–49.58, *p* = 0.078)	5.74 (0.69–47.49, *p* = 0.105)	6.65 (0.85–51.81, *p* = 0.070)	5.36 (1.24–23.19, *p* = 0.025)	4.61 (1.03–20.53, *p* = 0.045)	5.59 (1.29–24.18, *p* = 0.021)

HR, hazard ratio.

Cause of death was known in 18 patients (Table 1), and death was most commonly due to infection (*n* = 5, 27.8%) and cancer (7, 38.9%), as previously mentioned. In patients whose HCCs had either cell cycle or RTK pathway alterations, half or more of known deaths were due to cancer. In patients whose HCCs had WNT pathway alterations, only a third of known deaths were due to cancer. This did not reach statistical significance.

### 3.7 Validation of prognostic molecular features in a separate HCC cohort

The TCGA validation cohort is a common public resource in cancer genomics research (1, 30, 32). The publicly available TCGA cohort consisted of 372 patients. Alterations were again identified in the same three pathways, namely the RTK-RAS-PIK3 pathway in 12 (3.2%) tumors, the cell cycle pathway in 88 (23.7%) tumors, and the WNT pathway in 21 (5.6%) tumors. There were 11 tumors with cell cycle pathway alterations due to gene-level mutations in *CDKN2A* (two tumors) or *RB1* (nine tumors) with wild-type *TP53*. There were 7 (1.9%) tumors with alterations in two pathways. Cell cycle pathway alterations were again significantly associated with overall and progression-free outcomes in uni- and multivariable analyses (Supplementary Table S4 and Figures 2B,D). At the univariable level, cell cycle pathway alterations were more significantly associated with OS and progression-free survival (PFS) than *TP53* gene-level alterations alone (*p* = 0.002 vs. *p* = 0.008; *p* = 0.0017 vs. *p* = 0.014; respectively, Supplementary Figure S5). At the multivariable level, models including cell cycle pathway alterations vs. *TP53* gene-level alterations alone provided a significantly better fit to the OS and PFS data (*p* < 0.001 for each by ANOVA).

## 4 Conclusions

The increasing incidence and mortality rates of HCC (4) necessitate a review of current burden-based LT indications. Our study prospectively profiled the molecular tumor biology of a clinical HCC cohort post-LT, with sequencing results delivered to treating clinicians in a timely manner. We demonstrate that pathway-level alterations are strongly associated with post-LT outcomes. This real-world evidence suggests that tumor biology can directly inform transplant care and, with pre-LT testing, may eventually serve as a basis for revised transplant selection criteria.

Post-LT HCC outcomes were significantly associated with *TP53*-based gene- and pathway-level alterations. While *TP53* was the most prognostic gene, associations at the pathway level—involving related genes like *CDKN2A* and *RB1*—further reflected aggressive tumor biology. A multivariable analysis demonstrated that HCCs with cell cycle pathway alterations were associated with significantly lower OS and RFS and were characterized by a higher frequency of initial recurrence in the liver and lungs. The prognostic association between cell cycle pathway alterations and survival was externally validated in the TCGA HCC cohort, consisting more of resection cases (13, 30, 33). Given the prior observation that prognostic molecular features in colorectal liver metastases are shared across resectable and non-resectable cases (24), this replication in a non-transplant cohort confirms the broad prognostic utility of cell cycle pathway alterations in HCC, independent of the transplant setting.

Beyond cell cycle alterations, the RTK-RAS-PIK3 pathway was also altered in both our cohort and the TCGA series, a finding previously linked to adenocarcinoma-like HCC (1, 34). A large proportion of our patients showed no alterations in these pathways, which may reflect the limited sensitivity of a targeted sequencing panel. Interestingly, lower mutational burden has been paradoxically associated with enhanced antitumor immunity in HCC, a relationship warranting future investigation via complementary RNA sequencing and deconvolutional approaches (35, 36). Our findings align with the established classification of HCC into two major molecular subtypes (1–4, 11, 18, 35, 37)—proliferative (marked by mutations, cell cycle alterations, and an aggressive phenotype) and non-proliferative (characterized by mutations, WNT pathway alterations, and a less aggressive phenotype). This agreement redemonstrates that clinically meaningful tumor biology can be reliably identified using targeted sequencing, similar to its utility in breast cancer (38).

Timely availability of molecular data is critical for informing treatment decisions. In this series, molecular results were reported at a median of 27 days (IQR, 21−43) from LT. This rapid turnaround, achievable with targeted sequencing, demonstrates the feasibility of integrating HCC tumor biology into LT patient care. Targeted sequencing also offers the advantage of easy implementation and validation as an internal clinical platform. Molecular results ideally should be available within 2−4 weeks of initiating first-line therapy to prepare for second-line options upon progression (39, 40). Molecular profiling performed in this cohort was intended to determine clinical feasibility and association with outcomes. The molecular results did not directly inform patient care, neither for immunosuppression nor for adjuvant therapy.

The key strengths of this study are the cohort's ethnic diversity and its focus on LT, differing from other HCC molecular series that often include non-cirrhotic resection or biopsies of advanced stage patients. These factors may be associated with HCC driver gene alteration frequencies (19, 41). While post-transplant sequencing limits direct inference for pre-LT selection, this work offers insights into the clinical implementation of molecular profiling in the LT setting. Post-LT molecular data may be valuable for augmenting recurrence risk models and guiding emerging adjuvant therapies (42–44). This methodology is directly transferable to future pretransplant studies using solid or liquid biopsy to inform patient selection and neoadjuvant treatment, as endorsed by current HCC management guidelines (45).

This study has several limitations. First, the moderate sample size of this study, while comparable to that of other clinical HCC series (23), is smaller than that of retrospective sequencing studies that often lack clinical granularity (1). To our knowledge, this is the only post-transplant HCC sequencing series. Second, the reported gene-level mutation frequencies in this study are lower than those in larger prior studies (1, 15, 19, 21, 23). This may be due to the inherently lower sensitivity of the targeted DNA panel (which was not optimized for common HCC somatic alterations) or the use of neoadjuvant therapy (although not associated with specific alterations). Third, this study could not replicate the reported association between *TP53* mutations and Hepatitis B virus (HBV) etiology (1, 11, 19). Our sequencing approach could not detect oncogenic HBV-related mechanisms such as viral insertional mutations (14, 20, 46) or *IRF2* biallelic inactivation (11). Fourth, the low frequency of systemic therapy use prevented us from replicating known associations between pathway-level alterations and response to systemic treatments (21, 23). Also, while liver-directed or systemic therapies may exert selective pressure on tumor cells, potentially influencing the landscape of genomic alterations (47), our study design did not allow us to determine whether observed genomic alterations were present *de novo* or were enriched due to therapies. Fifth, our focus on genomic alterations did not allow for characterization of the tumor immune microenvironment, which is known to influence HCC biology (48). This is an area of a planned future study, including immunohistochemistry and RNA sequencing, and such a study may further improve our prognostic models. Moreover, as our patients largely adhere to our institution's clinical practice guidelines for immunosuppression by using tacrolimus and mycophenolate, we could not investigate the impact of distinct immunosuppression on outcomes. Finally, while the TCGA cohort provided external validation for our prognostic findings, it does not constitute a genuine transplant cohort.

In summary, we demonstrate that the molecular features of explanted HCC tumors are strongly associated with post-transplant recurrence and survival. Our findings establish that targeted sequencing methods can be implemented in a timely manner within clinical practice to inform patient care. Specifically, patients with tumors exceeding conventional size and number criteria, but lacking cell cycle pathway alterations, may potentially be considered safely for liver transplantation (LT). Future prospective, intention-to-treat studies integrating pretransplant molecular profiling via solid or liquid biopsy are warranted to refine LT selection criteria.

## Data Availability

Data will be available upon reasonable request made to the corresponding author.
