# Supplementary material for: Cell cycle pathway alterations predict outcomes post-liver transplantation for hepatocellular carcinoma
Source: Front Transplant. 2026 Apr 10;5:1758576. doi: 10.3389/frtra.2026.1758576 (PMC13106343; doi:10.3389/frtra.2026.1758576)
Supplement: Supplementary file 2 [file Table2.docx]

**Supplemental data**

**Supplementary tables**

**Table S1.** Clinical and pathological characteristics of patients with hepatocellular carcinoma undergoing liver transplantation stratified by use of molecular profiling.

**Table S2.** Gene-level mutations detected in hepatocellular carcinomas of patients undergoing liver transplantation, with numbers of carriers and annotations with prognostic and therapeutic implications.

^a^ For prostate cancer

^b^ For all solid tumors

^c^ For T-lymphoblastic leukemia/lymphoma

^d^ For oncogenic mutations in acute myeloid leukemia, chronic lymphocytic leukemia/small lymphocytic lymphoma, essential thrombocythemia, myelodysplastic syndromes, myeloproliferative syndromes, myeloproliferative neoplasms, primary myelofibrosis, therapy-related myeloid neoplasms

*ALK*, anaplastic lymphoma kinase; *APC*, adenomatous polyposis coli, *ATM*, ataxia telangiectasia mutated; *CDKN2A*, cyclin-dependent kinase inhibitor 2A; *CTNNB1*, catenin beta 1; *IDH2*, isocitrate dehydrogenase 2; *NOTCH1*, neurogenic locus notch homolog protein 1; *PIK3CA*, phosphatidylinositol 3-kinase catalytic subunit alpha; *PTEN*, phosphatase and tensin homolog; *RB1*, retinoblastoma 1; *SMAD4*, mothers against decapentaplegic homolog 4; *TP53*, tumor protein 53; *VHL*, von Hippel-Lindau tumor suppressor

**Table S3.** Demographics, clinical characteristics, and outcomes of patients undergoing liver transplantation for hepatocellular carcinoma stratified by WNT and RTK-RAS-PIK3 pathway alterations.

BMI, body mass index; d, days; ICU, intensive care unit; HCC, hepatocellular carcinoma; LT, liver transplantation; LRT, locoregional therapy; MELD, model for end-stage liver disease; TACE, transarterial chemoembolization; TARE, transarterial radioembolization

**Table S4.** Association of molecular subtypes and T stage with hepatocellular carcinoma outcomes in the TCGA cohort.

*CTNNB1*, catenin beta 1; HR, hazard ratio; *TP53*, tumor protein 53

**Supplementary figures**

**Figure S1.** Time in days from transplantation to pathology reporting (light blue) and molecular reporting (dark blue) per case.

**Figure S2.** Heatmap of clinical, pathological and molecular features ordered by Cell Cycle Pathway alterations. Features significantly associated with Cell Cycle Pathway alterations are denoted with asterix. For p-values, please see Table 1.

**Figure S3.** Clinical, pathological and molecular features differing significantly between tumors with intact and altered Cell Cycle Pathways. Differences are shown by absolute values (left) and by proportions (right), and by barplots (rows 1-4) and boxplots (row 5). For p-values, please see Table 1.

**Figure S4. Post liver transplant outcomes** **for 92 patients with hepatocellular carcinoma.** Overall (A & B) and recurrence-free (C & D) survival stratified by total number of gene-level mutations (A & C) and total number of pathway alterations (B & D).

**Figure S5.** Overall (A & B) and progression free survival (C & D) in the TCGA validation cohort stratified by *TP53* gene alterations (A & C) and cell cycle pathway alterations (B & D).
