# Supplementary figures and images for: Cell cycle pathway alterations predict outcomes post-liver transplantation for hepatocellular carcinoma
Source: Front Transplant. 2026 Apr 10;5:1758576. doi: 10.3389/frtra.2026.1758576 (PMC13106343; doi:10.3389/frtra.2026.1758576)

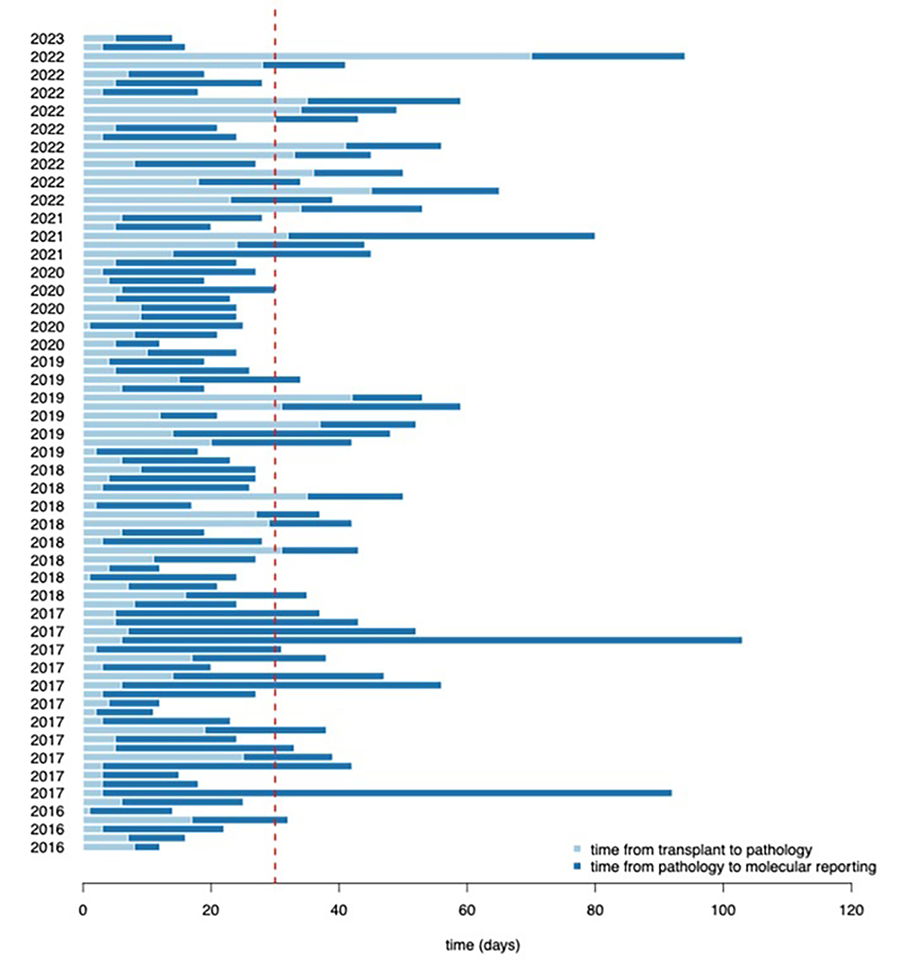

Supplement: Supplementary file 3 [file Image1.tif]

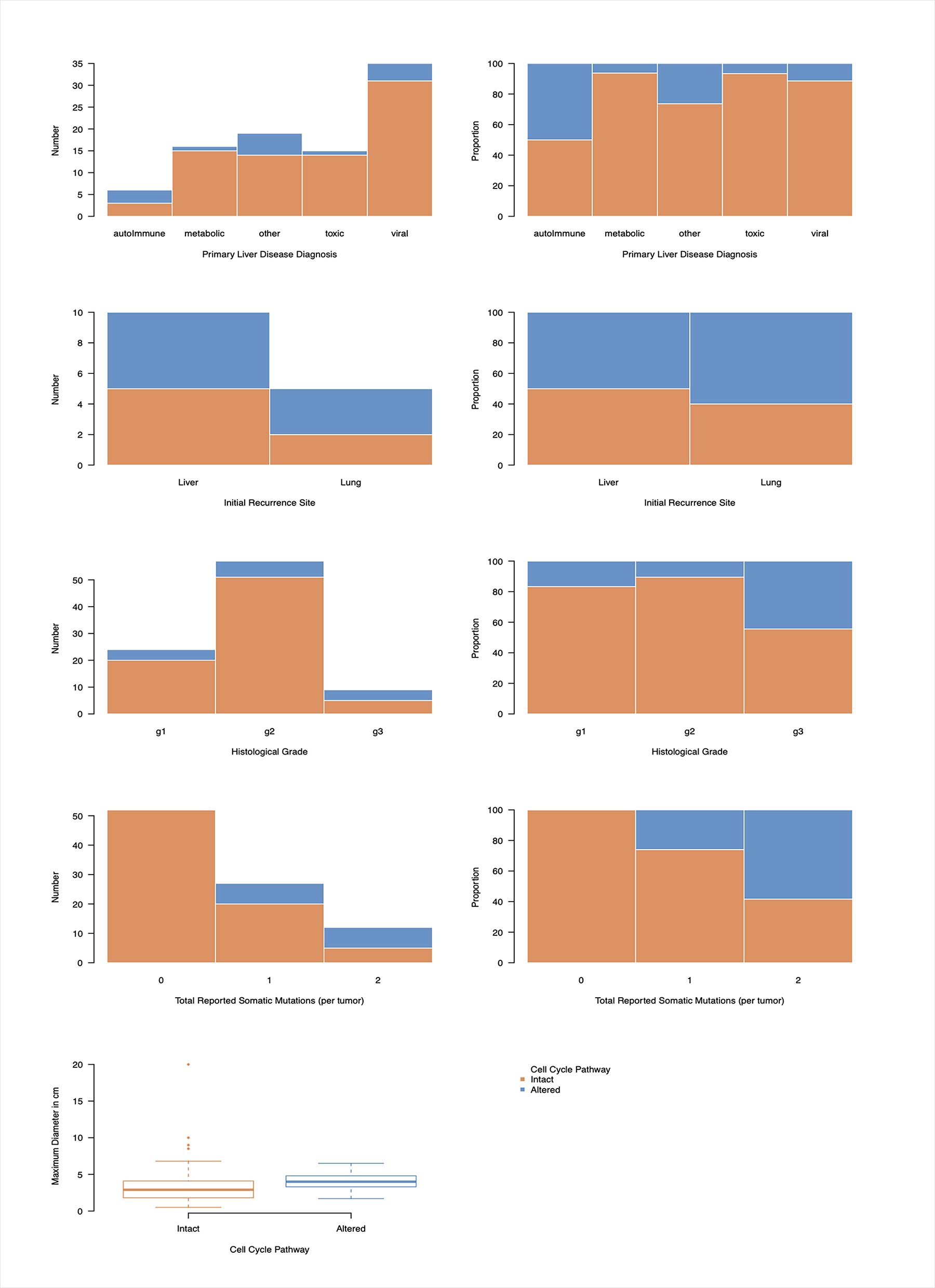

Supplement: Supplementary file 4 [file Image2.tif]

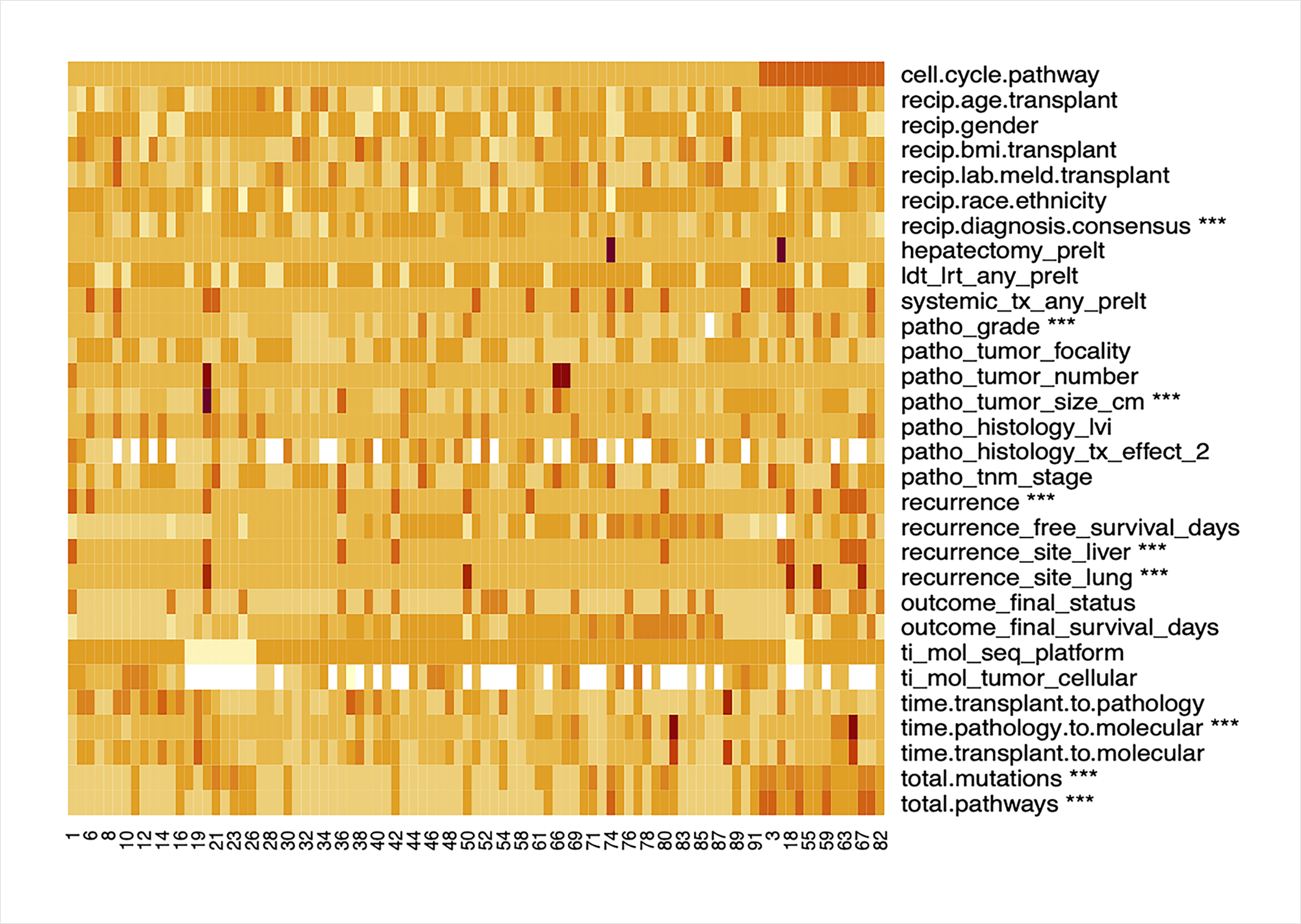

Supplement: Supplementary file 5 [file Image3.tif]

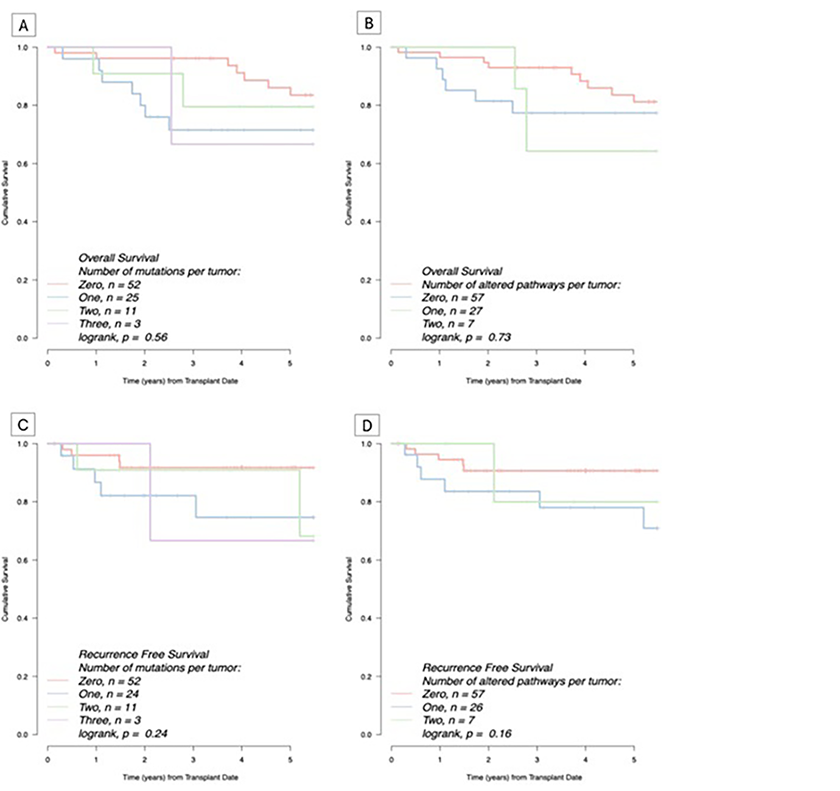

Supplement: Supplementary file 6 [file Image4.tif]

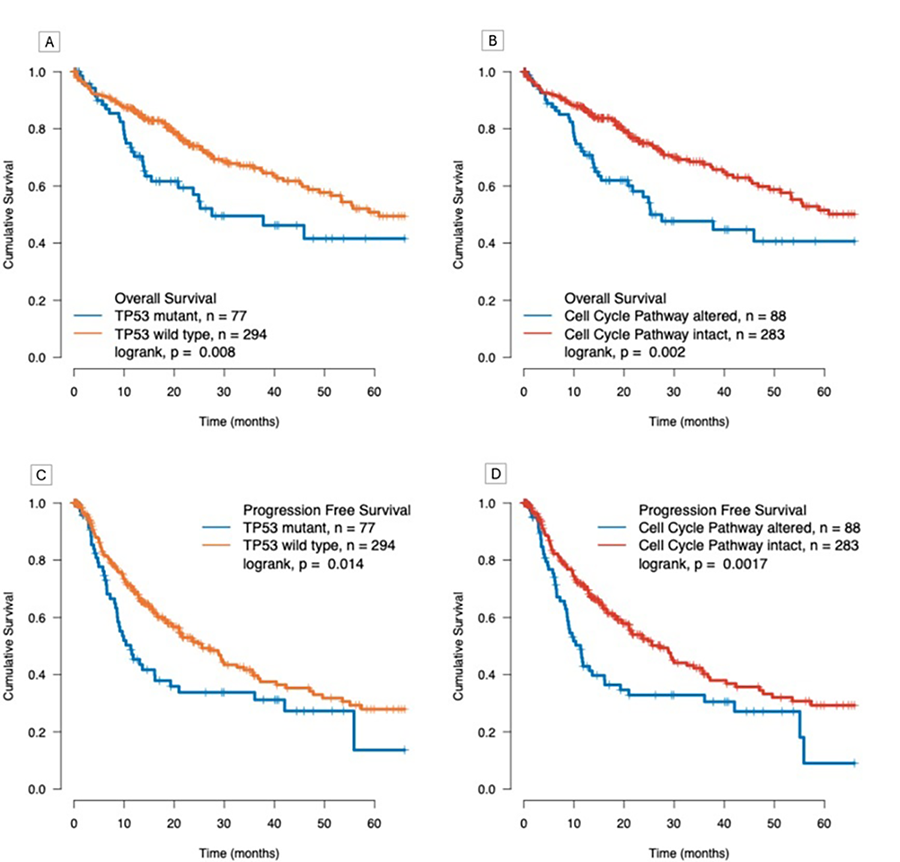

Supplement: Supplementary file 7 [file Image5.tif]
